# Monitoring the Performance of National Immunization Programs: Innovative Methodology and Tool for Countries’ Self-Assessment

**DOI:** 10.3390/vaccines14030258

**Published:** 2026-03-13

**Authors:** Sergio Loayza, Bertha Capistrán, Marcela Contreras, Martha Velandia, Daniel Salas

**Affiliations:** Special Program for Comprehensive Immunization, Pan American Health Organization, Washington, DC 20037, USAvelandiam@paho.org (M.V.);

**Keywords:** immunizations, assessment, expanded program on immunization, planning, budget, performance

## Abstract

**Background/Objectives**: In the context of its policy of “Reinvigorating Immunization as a Public Good for Universal Health,” the Pan American Health Organization (PAHO) developed a methodology and tool (MD-PAI) to help Member States of the Americas monitor and assess the performance of their national Expanded Program on Immunization (EPI) for each of the 13 technical components that make up the program. **Methods**: The MD-PAI was developed in several stages, including review of existing national EPI evaluation methodologies, selection and prioritization of questions for each of the 13 EPI components, piloting of the methodology, final calibration to ensure validity, completeness, reliability, standardization, usefulness, and usability across components and across countries, and publication in the four official languages of the PAHO. **Results**: The implementation of the MD-PAI enables countries to collect data, document lessons learned, develop action plans to close the most urgent gaps in the short and medium term and enforce the management of the EPI as part of the continuous improvement process. Since its introduction in 2023, fourteen countries in the Americas implemented the MD-PAI, using the results for their short- and medium-term planning and budgeting. Of the 13 components of the EPI, those that have performed best are political priority and planning and programming, while social communication is the component that reported the greatest number of gaps across countries. **Conclusions**: The PAHO has developed a methodology and tool to help countries to assess their EPIs to identify good practices, gaps and challenges, and develop an action plan to strengthen their programs. However, the impact of vaccination coverages and the epidemiology of vaccine-preventable diseases could take time.

## 1. Introduction

The Expanded Program on Immunization (EPI) was created in the region of the Americas in 1977 during the 25th Directing Council (DC) of the Pan American Health Organization (PAHO) with the aim of reducing morbidity and mortality from vaccine-preventable diseases such as diphtheria, pertussis, tetanus, measles, poliomyelitis, and tuberculosis [1,2]. Over the decades, coverage for vaccines against these six diseases (BCG, DPT3, polio3, MCV1 (Tuberculosis vaccine, Bacillus Calmette-Guérin (BCG); third dose of the vaccine against Diphtheria, Bordetella Pertussis, and Tetanus (DPT3); third dose of the oral or inactivated Polio vaccine (Polio3); and first dose of the measles-containing vaccine (MCV1))) rose to among the highest in the WHO regions, so that in the first 15 years of the new millennium, coverage exceeded 90% for all years and all these vaccines [3]. Thanks to these efforts, five of these diseases have been declared eliminated in the region: polio in 1994 [4,5], rubella and congenital rubella syndrome in 2015 [6], measles in 2016 and in 2024 [7,8], and neonatal tetanus in 2017 [9].

However, starting in 2013, according to WHO/UNICEF Estimates of National Immunization Coverage (WUENIC), the regional coverage rate for most antigens began to gradually decline, with polio3 being the first to fall below 90% in 2016, followed by MCV1 and DPT3 the following year. This situation exacerbated during the pandemic years, with BCG reaching 70% in 2020, polio3 reaching 80%, DPT3 reaching 81%, and MCV1 reaching 86% [3]. By 2021, the number of countries reporting more than 10% of children with no vaccine doses administered (i.e., zero-dose children) had doubled compared to 2010 [10].

In 2021, in response to this change in coverage trends and to support the regional implementation of Immunization Agenda 2030, the 59th PAHO DC approved the policy “Reinvigorating Immunization as a Public Good for Universal Health”. Member States were urged to adopt and implement the six strategic lines of action established by the policy [11]. These strategic lines of action, now part of the Regional Immunization Plan for the Americas 2030 (RIAP2030) [12], are: improve coverage monitoring and surveillance using digital intelligence; strengthen governance, political leadership, and financing of the EPI; strengthen human resource (HR) capacities; develop innovative communication approaches to build awareness, trust, and access to services; integrate the EPI into Primary Health Care (PHC) systems; and use scientific evidence to guide decision-making.

To support countries in achieving these objectives, the PAHO’s Special Program for Comprehensive Immunization (CIM) developed a methodology and tool for Monitoring the Performance of the EPI (with the Spanish acronym MD-PAI) that allows the rapid, efficient, and sensitive identification of gaps, good practices, and lessons learned in the implementation of the national EPI. The purpose of this assessment exercise is to support the development of short- and medium-term action plans to strengthen the vaccination actions.

Even though there are methodologies to monitor and evaluate the performance of an EPI, most of them are focused on the epidemiologic impact, global outcomes of the program (i.e., coverages) or performance in specific components of the EPI. Global agencies like the WHO, UNICEF, and Gavi have more comprehensive methodologies but generally use previously recollected data or their implementation requires an important mobilization of resources and time [13]. A specific concern that motivated the creation of a new methodology was the link between the results of the evaluation and the development of actions to improve those results. Maturity models (MMs), an evaluation framework coming from the informatic sector, were considered to facilitate this transition. The use of this MM in the health sector has been increasing over the last few decades to evaluate different processes at the health facilities level [14], but also in public health [15]. The assumption behind it is that to improve the performance, the processes of an organization transit from immature to mature states through a progressive and known road, giving a roadmap to continuous quality improvement [16].

**Figure 1:**
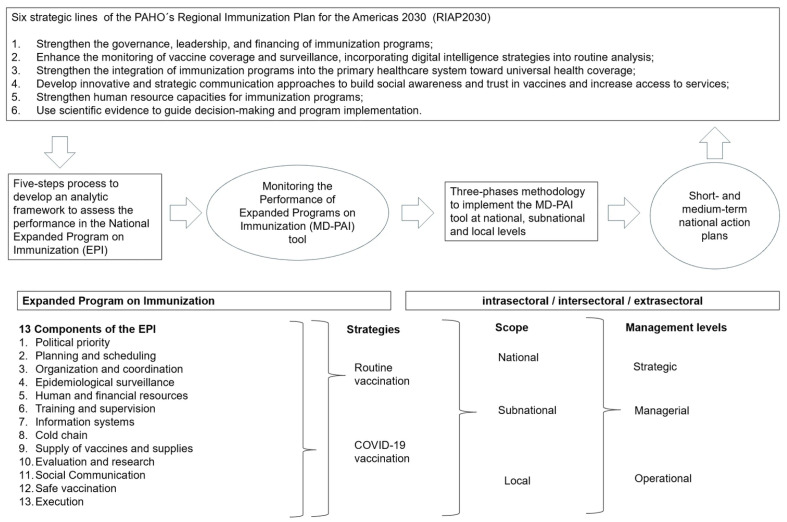
Basic outline for characterizing the process to build the MD-PAI tool, implementing it and to develop an action plan to address the six strategic lines of the Regional Immunization Plan for the Americas 2030 of the PAHO.

The objective of this article is to share the experience of the region on the MD-PAI introduction, a tool based on an MM, describing the five-step process that was used to develop the tool, the three-phase methodology for country implementation, and some of the results obtained in 14 countries of the region between 2023 and 2025 (Figure 1).

## 2. Materials and Methods

### 2.1. Tool Development

Five steps were used in the construction of the MD-PAI: (a) review of existing EPI evaluation methodologies and tools for the EPI and other health programs; (b) selection of topics or questions for each technical component of the EPI; (c) tool calibration; (d) piloting of the first version; and (e) publication and dissemination.

(a)  Review of existing EPI and other health program evaluation methodologies and tools

To define an analytic framework and topics to assess, the following PAHO/WHO evaluation tools and their implementation methodologies were analyzed:

• A Guide for Conducting an Expanded Program on Immunization (EPI) Review from the WHO [17]. Provided the foundational framework for comprehensive program evaluations.
• COVID-19 vaccine post-introduction evaluation (cPIE) guide from the WHO [18]. Informed the assessment of pandemic vaccine integration and emergency response.
• Methodology for the International Evaluation of the Expanded Program on Immunization (with the Spanish acronym EIPAI) from the PAHO [19]. Provided the primary categorical structure (13 EPI components).
• COVID-19 vaccine integration self-assessment tool from the WHO, UNICEF, and GAVI (unpublished version) [20,21]. Focused on the transition of COVID-19 vaccination into routine immunization services.
• Maturity Assessment Levels: Information Systems for Health IS4H-MAL, from the PAHO [22]. Directly informed by the maturity-level approach, the assessment is shifted from binary to a progressive scale.
• Joint External Evaluation (JEE) International Health Regulation [23], from the WHO. Served as a model for external evaluation of core public health capacities.
• States Parties Self-assessment annual report of International Health Regulation [24], from the WHO. Influenced the structure of self-reporting and maturity-based performance tracking.

The restriction to those specific resources, some of them already used in the region, was based on their comprehensive approach to an immunization process that includes all key domains. From these resources, questions were compiled across different components of the EPI.

(b)  Selection and prioritization of questions for each component of the EPI

Technical components of the EPI were defined, trying to be comprehensive and to communicate that, along with coverage goals, there are other factors to consider, and it would be necessary to involve more actors within and outside the Ministry of Health to ensure the success of the EPI. Within each component, the questions were subdivided depending on the administrative level they addressed (national, subnational, or local), the level of management they targeted (managerial, strategic, operational), and whether they required direct observation, field verification, or documentation (Figure 1). Within each category, they were prioritized according to the evaluation of PAHO CIM subject-matter experts as “essential”, “potential”, or “non-essential”.

Internal subject-matter experts (from CIM/PAHO) with knowledge and experience in the technical components and in the EPI of the region were defined. Iterative semi-structured interviews were done to prioritize the more relevant topic in every component and category, and to define, according to the evidence, the elements required to reach a high-level performance. The number of internal experts for components varied between one and three. The consensus came from an iterative process through a sequence of interviews and from prioritization topics to create the question and levels.

For each question, a 5-point scale was developed to assess the maturity of the specific item. This scale ran from 1 to 5, with level 1 being the lowest, indicating that the topic had only just been initiated by the national EPI, and level 5 indicating that the topic had been fully implemented and optimized (Table 1). This type of scale, which has been used in the monitoring tools of the International Health Regulations [23,24] and in the Maturity Assessment Levels: Information Systems for Health IS4H-MAL [22], allows for greater sensitivity and specificity in finding gaps, which makes it easier for countries to build more specific short- and medium-term action plans.

The construction of the levels was based on known technical recommendations made by the PAHO, WHO, or another group of experts. Also, an exhaustive search was conducted for policies and guidelines to support each question, in order to define what, in each topic, can be considered good practices or best performance (level 5).

(c)  Tool calibration

To improve the internal consistency of the methodology, the final phase of development was carried out by an external consultant (ACASUS), focusing on three objectives: validity and completeness, standardization of questions and levels, and internal reliability across EPI components and across administrative levels. Intra-component analyses were performed to ensure completeness (ensuring that all 128 topics accurately captured the full complexity of the EPI) and overlap between questions and levels within the component; between components to avoid repetition of topics and ensure comprehensiveness; between levels to ensure consistency and relevance to the scope of the question; and a review of each question to ensure internal consistency, temporal and topic specificity, objectivity, and measurability. The standardization of the MD-PAI tool focused on the consistency 5-point maturity scale, ensuring that level definitions were precise and that the “distance” between levels was consistent and interpretable across all topics. The wording of the questions and levels had to ensure consistency and facilitate understanding.

Finally, to maximize the reliability of the MD-PAI tool, the protocols and their annexes were completed, including templates for different processes and reports, and the tool’s interface was improved.

(d)  Pilot testing of the MD-PAI in Guyana and Guatemala

The MD-PAI was piloted in Guyana and Guatemala in April and July 2023, respectively. The objective of the pilot was to apply the tool using the implementation methodology to obtain feedback to improve it. In both cases, the questions and their maturity levels were presented to a group of EPI-related professionals at the national level, gathering feedback on comprehensibility, consistency, and relevance. The implementation in the pilots included a preliminary document review, local adaptation of the tool, a visit to a health facility, workshop methodology with different key actors and stakeholders for the application of the tool, and the development of an action plan. As part of the final calibration, key EPI professionals and a PAHO national consultant were interviewed by the external consultant, through a semi-structured instrument, to obtain opinions about compressibility of the 5-level scale, the pertinence and relevance of the questions, interphase of the tool, time and cost of the implementations, and methodology of the national workshops, subnational visit, and action plan development.

(e)  Publication and dissemination

Once the final version of the tool and methodology of the MD-PAI was ready, all documents developed in English were translated to the PAHO official languages (Spanish, French and Portuguese) and published on the website of the PAHO (https://www.paho.org/en/topics/immunization/performance-monitoring-tool-national-expanded-program-immunization, accessed on 22 December 2025).

The PAHO, through its country and sub-regional offices, encouraged health political authorities to evaluate their EPIs with the new methodology as part of their planning processes. The tool, methodology and the experience of the other countries where the MD-PAI had already been implemented were presented to the national EPI teams in regional meetings and webinars. Bilateral meetings were scheduled too, with the objective of familiarizing the process of implementation with the countries and solving doubts and concerns about the MD-PAI.

### 2.2. Implementation Methodology

**Figure 2:**
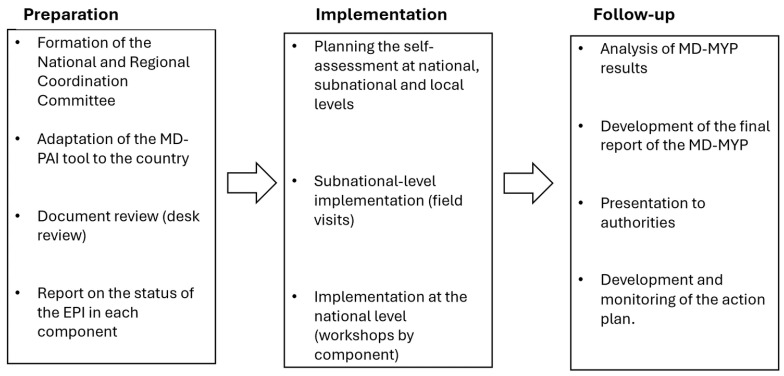
Phases and tasks of Performance Monitoring of National Immunization Programs.

The MD-PAI was developed as a voluntary documented self-assessment that can be conducted by the leadership of EPI national managers with secretariat support from the PAHO. It is based on the application of the MD-PAI tool, and it is implemented in three phases: preparation, implementation, and follow-up (Figure 2), each with clearly defined specific tasks and tools to facilitate the implementation (see Table 2).

The first phase begins with the formation of a National Coordination Team (NCT) whose functions include planning and executing the MD-PAI. The composition of the NCT is decided by the country, but it is recommended that professionals from the EPI and other units of the ministries of health and institutions involved in key EPI processes participate. The PAHO immunization focal point is also part of the NCT, as it is recommendable to hire a consultant to support all the phases. At the regional level, a Regional Coordination Team (RCT) accompanies the NCT during the different phases of implementation.

The adaptation of the MD-PAI tool, including the country’s own administrative nomenclature and other possible language differences, aims to facilitate its understanding by all participants. The document review, for its part, generates the evidence that supports the decision at each level for each topic, but also elements of the country context that explain these results. As a result of this first phase, a status report and a documentation repository on the EPI are expected to be available.

In the implementation phase, the aim is for the questions to be answered by the country teams at each level; however, only the performance levels achieved at the national level will be considered as an MD-PAI result. Subnational and local questions are used as verifiers, most of which are on the same topics consulted at the national level or are results of the main program processes.

To apply the MD-PAI tool at the subnational level, convenience sampling was used. The country is expected to select at least three subnational areas and, in each one, three local areas or districts, in order to select one health center for each (nine in total). The number of areas and centers will depend on the resources available and the feasibility of visiting them. The choice of these units is up to the country (randomness is not required for practical reasons), but it is recommended that a risk analysis be conducted to select those with high, medium, and low risks in the three subnational levels visited. The methodology used in the risk analysis is up to the country, but the measles risk assessment is available and recommended.

The national-level analysis is addressed through workshops, one for each component of the EPI. In each workshop, attendees, who are experts on the subject at different levels, discuss each of the questions, the level of performance of the EPI, and the evidence that supports it. The methodology encourages the participation of experts from both the health sector and other sectors (education, customs, economics), as well as from the Ministry of Health and other institutions (national reference laboratory, private facilities, social security), in order to enrich the discussion and counteract the possibility of potential overvaluation bias. It also encourages participants to analyze the reasons why the maximum level of performance has not been achieved in certain areas. The decision on the level of performance in each area is defined by group consensus among the participants, and the mechanism to get consensus must be defined by the country too. Members of the NCT, preferably more than one, are selected to take notes of the level reached in every question and the most relevant elements of the discussion. Considering that the result of the MD-PAI should feed an action plan, it is recommendable for a participant to declare any conflict of interest.

The MD-PAI provides averages and medians by component at the national and subnational levels, in tables and spider charts, for the regular program and COVID-19 vaccination separately. It also provides a dashboard output for both levels, with averages by component and subcomponent, indicating their relative weights.

Finally, in the follow-up phase, a final report on the implementation of the MD-PAI is prepared, the results are discussed with health authorities, and an action plan is developed to enable progress in the short and medium term on the topics prioritized by the country.

For each of these phases and activities, the methodology includes annexes with documents or templates to facilitate their implementation (Table 2).

## 3. Results

### 3.1. MD-PAI Tool

From the instruments reviewed, 744 questions were collected. After the prioritization process, 109 questions for the national level, 67 for the subnational level, and 20 for the local level were selected, addressing 13 components and 128 topics related to the EPI (Table 3). In addition, at the national and subnational levels, 18 and 3 questions, respectively, were focused on the integration of COVID-19 vaccination into the routine program.

The questions were organized by components in an Excel file that helps countries to collect information during the implementation of the MD-PAI. Every question is structured as a standard addressing a specific process, document or practice, the time to consider in the evaluation, and the elements that are considered relevant in its implementation. In the same line of the Excel sheet, the tool shows the levels from 1 to 5, where progressively, the elements are added following the MM. Then, every question is different, bringing five specific scenarios to be selected during the workshops. The participants of the workshop must define which scenario represents which EPI situation.

### 3.2. Implementation of the MD-PAI

Fourteen countries in the region have already implemented the MD-PAI. Guatemala, Guyana, and Saint Vincent and the Grenadines used the first version of the MD-PAI in 2023. In 2024, Brazil, Grenada, and Paraguay joined, followed in 2025 by Honduras, Ecuador, Jamaica, Peru, Chile, Dominican Republic, Dominica and Bolivia. Other countries of the Americas have shown interest in doing so during 2026.

Five countries implemented the MD-PAI in the context of the Joint External Evaluation of International Health Regulation core capacities. This represents a strategic alliance between the CIM/PAHO and the Department of Health Emergencies at regional level, to strengthen immunization as a component of the response to epidemics and take advantage of the will of the country to be evaluated.

All countries that have done the MD-PAI developed action plans for the next few months, the next year or a longer period (3 to 5 years). Most of the action plans were considered operational; however, some were more strategic. The planning perspective was up to the country.

Through an aggregate analysis of the results of the 14 countries that have implemented the MD-PAI, the two components with the highest ratings were political priority and planning and programming, and the lowest performance was social communication and demand generation. Topics like the use of different strategies to increase coverage or develop intersectoral coordination mechanisms proved to be well developed in every country. On the contrary, there are not common topics in the lower levels. There is also considerable variability within and between countries in the performance inside the components, showing that the EPIs do not develop with a unique pattern.

The time that it takes to implement the MD-PAI has been different among countries for internal reasons. The protocol recommends 16 weeks from the formation of the National Coordination Team to development of the complete three phases. Most of this time is dedicated to the preparation phase, as the implementation of the tool, at both the national and subnational levels, takes one week each.

The cost of the process varies between countries, and it is primarily related to the consultant contract, the number and accessibility of the subnational level visited, and the development of national workshops (i.e., meeting format, place, number of attendees). Annex 6 helps countries to estimate the cost of the M-PAI.

## 4. Discussion

To improve their performance, national EPI programs need to maximize their management capacities. The PAHO recognizes this complexity of immunization programs and proposes, in the context of RIAP2030, a tool and methodology for evaluating the performance of the EPI that facilitates the analysis of each component by countries and links it to actions to improve it.

A systematic review found, in the gray (65%) and peer-reviewed (35%) literature, 20 monitoring and evaluation resources measuring National Immunization Programs’ performance, impact and outcomes that have been published after 2000 [16]. Most of them were from global agencies like the WHO, Gavi and USAID, or related to specific programs of these agencies, like the Global Polio Eradication Initiative. From these resources, 631 different indicators were recognized, most of them (72.9%) addressing the performance of the EPI in its different components, and the others evaluating the impact or the global results of the program. There is an important coincidence between the topics included in these resources and the MD-PAI; however, great differences in the scale of the indicators, the source of the data (many using the WHO–UNICEF Joint Reporting Form on Immunization), and the participation of the EPI professionals were found.

The use of a maturity model in an immunization program at national level is an innovative approach, focusing on the quality of the process more than in the results of them and allowing them to put in knowledge of the elements necessary to reach the maximum level of performance. In a health care context, maturity models have been used to assess and improve different processes and technologies in specific settings [14]. A five-level maturity scale increases the sensitivity of the assessment to detect specific gaps compared to dichotomous scales. The premise behind the MM is that the development of functional areas is through progressive levels of performance, allowing planned actions to reach the next level in a more realistic way [25].

In a two-year period, 14 of the 35 PAHO’s member states have already implemented the methodology, including some of the biggest and smallest countries of the region, with centralized and decentralized EPIs. The fourteen countries have led to the development of short- and medium-term action plans tailored to their specific needs, with varying degrees of operational or strategic depth. This flexibility has proven essential for accommodating the diverse contexts and capacities across the region. We expect that more countries will implement the MD-PAI over the next few years. It is a country’s decision, and it is important, for the achievement of the objective, to be fully convinced of the worth of the assessment.

One of the main strengths of the MD-PAI is its participatory, consensus-driven approach. By engaging stakeholders from multiple administrative levels and sectors—both within and outside the health system—the tool builds a shared understanding of program performance and strengthens national ownership of results. Each MD-PAI exercise has involved representatives not only from immunization programs but also from other health ministry units and sectors such as education, finance, information systems, social protection, and communication. This intersectoral collaboration underscores the recognition of immunization as a public good that requires coordinated efforts across government and society. Such broad engagement has enriched the exchange of best practices, lessons learned, and challenges, enabling a more comprehensive interpretation of findings and consensus on next steps. It also fosters deeper analysis of the factors underlying successes and gaps, potentially enhancing commitment to the agreed action plan.

Although the subnational and local results are not considered in the MD-PAI results, the information extracted from these levels could be relevant in the discussion as evidence of gaps or good practices. It also allows operative levels to become involved in the exercise rather than just strategic and managerial. A probabilistic sample from the subnational level could decrease the risk of selection bias, be more representative and the results generalizable to that level in the country, but till now, no country has implemented it. However, it is possible that in some countries, the number of units to be visited could increase to improve the statistical significance, increasing the cost of implementation. Also, specific considerations regarding the type of sampling should be defined in each country.

Given that the MD-PAI is a self-assessment, there is a risk of overvaluation by the country if it is perceived as a judgment or if there are political expectations regarding its results. To counter this inherent risk in self-assessments, the MD-PAI incorporates some mitigation strategies like the requirement of evidence, which means that no performance level could be assigned without supporting evidence from the desk review or the verification from subnational/local site visits. The intersectoral triangulation (the methodology requires participation from experts in education, customs, finance, and social protection) provides external technical pressure to ensure that health staff assessments are realistic. Moreover, the presence of external consultants and PAHO focal points serves as a neutralizing influence, encouraging participants to analyze the underlying causes of gaps.

The use of averages in ordinal scales as a description of the component level, a debatable issue, has the pragmatic objective of visualizing and communicating the existence of gaps. This decision forces us to assume equal distance between levels and equal relevance of the questions. However, the tool gives medians and distribution tables too. The use of an ordinal scale presented as numerical has skewed the exercise toward the quantitative, even though the analysis itself is more qualitative in nature (as a public health intervention). For example, the average has led to discussions about the importance of decimals or, in other cases, about the validity of rounding up or down. For the other side, the median, it could be less intuitive, and it could hide gaps.

The impact that the MD-PAI will have on vaccination coverage should be evaluated over time. The strengthening in planning, organization, coordination, vaccine supply and demand generation should impact on the levels of protection against vaccine-preventable diseases. However, this impact could take time if we consider that it requires planning, implementing and monitoring, and process, budgets and human resource skills transformations. Nevertheless, the performance of an EPI is much more than just coverage, which is one of the lessons and messages that has emerged in countries that have applied the methodology. The true meaning of the coverage supposes good practice of registering and monitoring, coherence with the results of the surveillance, and must be understood in the context of the integrity of a cold chain, proper administration technique, and the certainty that immunization is being done safely.

## 5. Conclusions

The PAHO has developed a methodology and tool to help countries to assess their EPIs to identify good practices, gaps and challenges, and develop an action plan to strengthen their programs. Beyond its technical functions, the MD-PAI has also contributed to strengthening governance, accountability, and the culture of continuous improvement within national programs. In several countries, the results have been used to inform national planning processes, budget negotiations, and requests for technical cooperation. The tool has also fostered synergies with broader health system strengthening efforts, including digital transformation, data quality initiatives and alert and response systems for the International Health Regulations. In the coming years, the improvement in the protocols and tools, including new technologies, could ease the implementation of the MD-PAI to include more countries and shorten the processes. The consolidation of MD-PAI results across countries will allow regional analysis of trends, identification of common bottlenecks, and prioritization of technical cooperation.

## 6. Patents

ISBN: 978-92-75-12876-3 (PDF) ISBN: 978-92-75-12877-0 (Print) © Pan American Health Organization, 2025. Some rights reserved. This work is available under the Creative Commons Attribution-NonCommercial-ShareAlike 3.0 IGO license (CC BY-NC-SA 3.0 IGO).

## Figures and Tables

**Table 1:** Operational definitions for the development of performance levels.

Level	Definition	Characteristic
1. Initial	Scarce practices	Desirable elements to have, comply with, or perform are insufficient or unavailable
2. Managed	Repeated practices	Desirable elements to have, comply with, or perform that are available have completeness issues, need to be updated, or are not appropriate for use
3. Defined	Competency-based practices	Desirable elements to have, comply with, or perform that are available are routinely implemented, but there are biases or problems
4. Quantitatively managed	Measurable practices	All desirable elements to have, comply with, or perform are available and complete, reliable, and up to date. Some biases/problems are known and can be improved
5. Optimized	Practices based on continuous improvement	All desirable elements to have, comply with, or perform are available, up to date, and implemented consistently

**Table 2:** List of MD-PAI annexes.

Category	Number	Name	Type
Summary documents	A	Summary of annexes	Document
	B	National MD-PAI Protocol	Document
	C	Presentation to MD-PAI Member States	Presentation
	D	Terms of reference for consultants	Document
	E	Introductory presentation for consultants	Presentation
	F	Schedule of activities	Spreadsheet
Phase 1: Preparation	1	Presentation to introduce the MD-PAI to the NCT	Presentation
	2	Functions and responsibilities of the NCT and RCT	Document
	3	List of recommended documents for document review	Spreadsheet
	4A	Template for Situation Analysis Report	Document
	4B	Template for Situation Analysis Presentation	Presentation
	5	Guidelines for adapting the MD-PAI tool	Document
Phase 2: Implementation	6	MD-PAI budget spreadsheet	Spreadsheet
	7	Criteria for selecting subnational areas	Document
	8A	Guidelines for implementation of the MD-PAI at the subnational level	Document
	8B	Introductory presentation for the subnational level	Presentation
	9	Selection of workshop participants	Spreadsheet
	9b	List of participants and participation sheet for national workshops	Spreadsheet
	10	Agenda for national workshops	Document
	11	Guidelines for conducting national workshops	Document
	12	Opening Ceremony—Presentation of the MD-PAI	Presentation
	13	Components Workshop—MD-PAI Presentation	Presentation
	14	Question format	Presentation
	15	Gold standard for components	Spreadsheet
Phase 3: Follow-up	16	Final monitoring report—spreadsheet	Spreadsheet
	17	National action plan	Spreadsheet

MD-PAI, Performance Monitoring Expanded Program of immunization; NCT, National Coordination Team; RCT, Regional Coordination Team.

**Table 3:** Components and topics included in PAI performance monitoring.

Topics by Component	Level	Topics by Component	Level
**1. Political priority**		**7. Information systems**	
Legal framework for planning and operation	N	Information systems guidelines	N
Accountability	N	Vaccination record	N-L
Lifetime policy	N	ICT infrastructure diagnosis	N
EPI participation in decision-making	N	Integration COVID-19 and regular vaccination registry	N
International participation in immunization issues	N	Analysis of coverage and gaps	N-SN
Policy with COVID-19 operations financing	N	Equity analysis	N
**2. Planning and programming**		Monthly reports by facilities	N
Multi-year strategic plan for the EPI	N	Data quality analysis	N
EPI technical standards manual	SN-L	VPD coverage and surveillance situation room	SN
Use of strategies in routine vaccination	N	Integration of COVID-19 vaccination into PAI records	N
Criteria for introducing vaccines	N	Evaluation of COVID-19 coverage integration	N
Denominator data sources	N-SN-L	Monitoring of COVID-19 vaccination in high-risk groups	N
Assignment of areas of responsibility for facilities	N-SN	**8. Cold chain**	
Implementation and monitoring annual plan	N-SN	Cold chain regulations	N-L
Analysis of the situation of the PAI	N	Cold chain inventory	N
Strategies to reach under-vaccinated populations	N-SN-L	Maintenance and replacement plan	N-SN
Microplanning guidelines	N-SN-L	Critical variables in the vaccine management system	N
Alignment of planning with other programs	N	Cold chain temperatures	N-SN-L
Vaccination strategies for vulnerable populations	SN	Supply chain oversight	N
Integration of COVID-19 operations into the EPI	N	Cold chain monitoring	N-SN-L
Integration COVID-19 operations with other programs	N-SN	Resources for additional vaccine transportation	N
Target populations for COVID-19 vaccination	N	Vaccine storage at the airport	N
COVID-19 vaccination coverage for priority groups	N	Problem reporting protocols for revolving fund	N
COVID-19 vaccination strategies	N	Ultra-low-temperature chain capacity	N
**3. Organization and coordination**		Subnational storage capacity	SN-L
EPI organizational chart	N-SN	Vaccine loss	SN-L
National Technical Coordinating Committee	N-SN	Cool boxes and ice packs	SN
NITAG structure	N	Knowledge of vaccine thermal tolerance	L
Functioning of NITAG	N	Vaccine shortages	SN-L
Mechanisms for intersectoral coordination	N-SN	**9. Vaccine supply**	
External participation in vaccination operations	N-SN	Annual vaccine demand planning	N
Prioritization of high-risk areas	N	Basic functions of the NRA	N
International coordination for border activities	SN	Introduction of vaccines through the Emergency Use Listing or WHO Prequalification	N
Social security coordination	SN	Procedures for batch release	N
Private health sector coordination	SN	Stabilization reserves	SN
COVID-19 vaccine integration intersectoral group	N	**10. Evaluation and research**	
**4. Epidemiological and laboratory surveillance**		EPI evaluation and follow-up meetings	N-SN
VPD surveillance protocols	N-SN-L	Vaccination coverage surveys	N-SN
Annual VPD surveillance training plan	N-SN	Operational research	N
Inclusion of private laboratories in the surveillance	N	Post-vaccine introduction studies	N
Supplies for national virology laboratory	N	**11. Social communication and demand generation**	
Opportunity for polio and measles results	N	Assessment of acceptability and barriers to vaccination	N
National bacteriology laboratory supplies	N	Demand generation and communication plan	N-SN
Opportunity for bacteriology results	N	Communication plan resources	N
Reporting units	N-SN	Communication plan and community participation	N
Surveillance reporting rate measles/rubella	N	COVID-19 communication plan	N
Measles and rubella surveillance quality indicators	N	COVID-19 vaccine acceptability and barriers assessment	N
AFP surveillance notification rate	N	Orientation session on the EPI	SN
Adequate samples in AFP surveillance	N	**12. Safe vaccination**	
Measles and polio risk assessment	N-SN	ESAVI Manual	N-L
Rapid response teams	N-SN	ESAVI Nominal Database	N
Measles surveillance reporting tool	N	National Vaccine Safety Committee	N
Active finding of measles/polio cases	SN	Severe ESAVI investigation registration form	N
Measles and polio notification form	L	Programmatic error notification system	N
**5. Human resources and financial management**		Vaccine safety communication plan	N-SN
Source of vaccine funding	N	National training plan for ESAVI surveillance	N
Budget security and flexibility	N	Publication of ESAVI report	N
Fund release process	N-SN	Vaccine safety guidelines, safe injection, waste management	N-L
Budget planning	N-SN	Hazardous medical waste disposal standard	N
Resources for purchasing COVID-19 vaccines	N	Contracts for hazardous waste disposal	N
HR adequacy	N-SN	Multi-dose vial policy	N-SN-L
Human resources strategy	N	Educational material on safe injection and waste disposal	N
HR plan	N	Training on ESAVI surveillance	SN
Availability of funds for COVID-19 vaccination	N-SN	Training on programmatic error surveillance	SN
**6. Training and supervision**		**13. Execution**	
National training plan	N-SN	The items of this component are included in the other components but evaluated at local level	
Quality of the national training plan	N		
Training plan for vaccine introduction	N		
Subnational training over the last 24 months	N		
National Supervision Plan	N-SN		
Supervision visits at the subnational level	N-SN-L		
COVID-19 vaccination training	N		
Training on integrating COVID-19 vaccines into the EPI	N		
COVID-19 vaccination supervision	N		

N: national level; SN: subnational level; L: local level; NITAG: National Technical Advisory Group; HR: human resources; ESAVI: Events Supposedly Attributable to Vaccination or Immunization; VPD: vaccine-preventable diseases; WHO: World Health Organization; ICT: Information and communication technologies; EPI: Expanded Program on Immunization; NRA: National Regulatory Authority; AFP: Acute flaccid paralysis.

## Data Availability

Protocols and other document and file are available on the PAHO website: https://www.paho.org/en/topics/immunization/performance-monitoring-tool-national-expanded-program-immunization (accessed on 22 December 2025).

## Abbreviations

The following abbreviations are used in this manuscript:

AFP	Acute flaccid paralysis
BCG	Tuberculosis vaccine, Bacillus Calmette-Guérin
CIM	PAHO’s Special Program for comprehensive Immunization
cPIE	COVID-19 post-Introduction evaluation
DC	Directing Council PAHO
DPT3	Third dose of the vaccine against diphtheria, Bordetella pertussis, and tetanus
EIPAI	International Evaluation of the Expanded Program on Immunization
EPI	Expanded Program of immunization
ESAVI	Events Supposedly Attributable to Vaccination or Immunization
HR	Human resources
ICT	Information and communication technologies
MCV1	First dose of the measles-containing vaccine
MD-PAI	Performance Monitoring Expanded Program of immunization
MM	Maturity model
NCT	National Coordination Team
NITAG	National Technical Advisory Group
NRA	National Regulatory Authority
PAHO	Pan American Health Organization
PHC	Primary Health Care
Polio3	Third dose of the oral or inactivated polio vaccine
RCT	Regional Coordination Team
RIAP2030	Regional Immunization Plan for the Americas 2030
VPD	Vaccine-preventable diseases
WHO	World Health Organization
WUENIC	WHO/UNICEF Estimates of National Immunization Coverage
