# Correction: Loayza et al. Monitoring the Performance of National Immunization Programs: Innovative Methodology and Tool for Countries’ Self-Assessment. *Vaccines* 2026, *14*, 258

**DOI:** 10.3390/vaccines14050433

**Published:** 2026-05-12

**Authors:** Sergio Loayza, Bertha Capistrán, Marcela Contreras, Martha Velandia, Daniel Salas

**Affiliations:** Special Program for Comprehensive Immunization, Pan American Health Organization, Washington, DC 20037, USAcontrermar@paho.org (M.C.); velandiam@paho.org (M.V.);

The authors would like to make the following correction to this published paper [1].

Due to a change in the grant used to finance the publication item, the authors would like to update the Funding statement in this publication [1]. The original version is as follows:

**Funding:** In partnership with the Government of Canada and through the project on “Improving Equitable Access and Vaccination Coverage against COVID-19”, PAHO was able to collect the findings that are included in this article.

It should be updated to the following:

**Funding:** In partnership with the Government of Canada and through the project on “Improving Equitable Access and Vaccination Coverage against COVID-19”, PAHO was able to collect the findings that are included in this article. Additionally, this publication was supported by the Grant or Cooperative Agreement Number, NU66GH002176, funded by the Center for Disease Control and Prevention. Its content is solely the responsibility of the authors and does not necessarily reflect the official views of the Centers for Disease Control And Prevention or the Department of Health and Human Services.

The authors state that the scientific conclusions are unaffected. This correction was approved by the Academic Editor. The original publication has also been updated.

## References

[1] Loayza S., Capistrán B., Contreras M., Velandia M., Salas D. (2026). Monitoring the Performance of National Immunization Programs: Innovative Methodology and Tool for Countries’ Self-Assessment. Vaccines.

